# Sex-specific transcriptional regulation in lung macrophages during sub-acute *Mycobacterium tuberculosis* infection

**DOI:** 10.1128/spectrum.01790-25

**Published:** 2025-11-12

**Authors:** Dhanya Dhanyalayam, Hariprasad Thangavel, Kezia Lizardo, Jigar V. Desai, Jyothi F. Nagajyothi

**Affiliations:** 1 Center for Discovery and Innovation, Hackensack Meridian Health, Nutley, New Jersey, USA; University of Nebraska Medical Center, Omaha, Nebraska, USA

**Keywords:** *Mycobacterium tuberculosis*, sex-specific immune regulation, macrophages, transcriptomic profiling, host-pathogen interactions, AKT-IFN-γ signaling, IL-6 signaling, Immunoglobulin Heavy Constant Gamma 1 (Ighg1), autophagy, pulmonary infection

## Abstract

Biological sex significantly influences susceptibility to tuberculosis (TB), with males typically experiencing a greater disease burden than females. However, the mechanisms underlying sex-specific immune regulation during TB pathogenesis remain incompletely understood. In this study, we examined sex-based differences in pulmonary immune cell composition, inflammatory signaling pathways, and macrophage transcriptomic responses in C57BL/6 mice infected with the *Mycobacterium tuberculosis* (*Mtb*) HN878 strain during the sub-acute stage of infection. Our results show that female mice had significantly lower pulmonary *Mtb* burdens and enhanced protein kinase B (AKT)–interferon gamma (IFN-γ) signaling, which is associated with autophagy, lysosomal activation, and effective intracellular bacterial clearance. In contrast, male mice exhibited higher bacterial loads and elevated IL-6 signaling, a pathway linked to exacerbated inflammation and impaired pathogen control. Histological analysis revealed greater lymphocytic aggregation in female lungs, despite comparable levels of pulmonary macrophages between sexes. Importantly, transcriptomic profiling of lung macrophages uncovered distinct sex-specific gene expression patterns, wherein female macrophages upregulated genes involved in tissue remodeling, phagocytosis, autophagy/lipophagy, and cell survival, whereas male macrophages showed enrichment of genes related to pro-inflammatory and adaptive immune responses. Notably, male macrophages expressed higher levels of *Ighg1* (Immunoglobulin Heavy Constant Gamma 1), suggesting a potential sex-dependent modulation of humoral immunity during TB. Together, these findings demonstrate that biological sex shapes immune cell programming and host-pathogen interactions during TB, underscoring the importance of incorporating sex as a biological variable in TB research and therapeutic development.

**IMPORTANCE:**

Men and women often respond differently to diseases like tuberculosis (TB), with men typically facing more severe illness, though the underlying reasons are unclear. To this end, our study investigated how male and female mice combat TB infection at a cellular and molecular level. We discovered that female mice controlled TB more effectively, as their immune systems activated specific pathways to break down and clear bacteria efficiently. Conversely, male mice showed higher bacterial loads and triggered more inflammatory, yet less effective, immune responses. Crucially, despite similar numbers of key immune cells (macrophages) in the lungs, their functional responses differed significantly by sex. These findings underscore that biological sex profoundly impacts the immune system’s fight against TB, paving the way for more personalized treatments and improved outcomes for all.

## INTRODUCTION

Tuberculosis (TB), caused by *Mycobacterium tuberculosis* (*Mtb*), remains a major global health challenge and a leading cause of infectious disease-related mortality. Its persistence is driven by a range of factors, including drug resistance, HIV co-infection, rising rates of metabolic syndrome, and social and economic disparities—particularly in low- and middle-income countries. Diagnostic limitations, inadequate treatment options, and the impact of stigma and discrimination further complicate TB control efforts. Interestingly, epidemiological studies consistently show that TB incidence is higher in males, who are approximately 1.6 times more likely to develop active disease than females (1, 2). This disparity has been attributed to a combination of environmental and sociocultural factors, as well as to physiological differences, such as variations in hormonal regulation and adipose tissue distribution between the sexes (3, 4). Furthermore, immune control of infecting bacilli may differ between the sexes, as suggested by our study, demonstrating that male mice infected with the hypervirulent *Mtb* HN878 strain exhibit increased lung pathology and *Mtb* burden by 30 days post-infection (DPI) relative to female mice. This was accompanied by reduced leukocyte infiltration, elevated CD8^+^ T-cell responses, increased IL-6 levels, and altered lipid metabolism (5). Other studies have also reported sex-specific immune differences, such as impaired B cell follicle formation and greater inflammatory damage in male mice infected with *Mtb* strains H37Rv or HN878 (6, 7). Nevertheless, the mechanisms underlying sex-specific immune regulation during TB pathogenesis remain poorly understood.

Macrophages play a central role in TB pathogenesis—not only as primary host cells harboring *Mtb* but also as regulators of innate and adaptive immune responses. Macrophages produce cytokines and chemokines to promote antimicrobial activity, orchestrate T and B cell responses, and facilitate granuloma formation (6, 8). However, *Mtb* has evolved mechanisms to subvert macrophage function, promoting immune evasion and long-term persistence. In particular, *Mtb* can promote macrophage M2 polarization and formation of foamy (lipid-laden) macrophages, which are associated with reduced ability to restrict *Mtb* growth and survival (9, 10). On the other hand, excessive M1 polarization can also have adverse effects as it can lead to tissue-damaging inflammation via uncontrolled cytokine release and T-cell hyperactivation (11). Macrophage cellular processes, such as phagocytosis, autophagy, lysosomal degradation, and lipophagy, are critical for bacterial clearance and tissue homeostasis (12, 13). However, despite the centrality of macrophages in TB defense, their sex-specific transcriptional programming and functional reprogramming in response to *Mtb* infection have remained underexplored.

We hypothesized that biological sex influences the transcriptional and functional responses of lung macrophages during pulmonary *Mtb* infection, contributing to differences in inflammation, pathogen clearance, and tissue recovery during TB. To test this hypothesis, we infected male and female C57BL/6 mice with *Mtb* HN878 via aerosol and examined the lungs at 30 DPI (sub-acute stage of infection). We assessed bacterial burden, immune cell infiltration, inflammatory signaling, and markers of cell survival. Additionally, we performed transcriptomic profiling of lung macrophages to evaluate sex-dependent differences in genes regulating inflammation, phagocytosis, autophagy/lipophagy/lysosomal function, and cellular survival. Our findings demonstrate that female mice had significantly lower *Mtb* burden and exhibited enhanced AKT–IFNγ signaling, which promotes autophagy and survival pathways. In contrast, male mice showed higher bacterial loads, increased IL-6-driven inflammatory responses, and elevated expression of genes linked to both innate and adaptive immunity. Transcriptomic analysis revealed that female macrophages preferentially upregulated genes involved in tissue repair, antimicrobial defense, and cellular homeostasis, whereas male macrophages displayed a more pro-inflammatory and immune-activated gene signature, including elevated expression of *Ighg1*, a marker of humoral immune activation. In summary, our study identifies macrophage transcriptional reprogramming as a hallmark of sex-specific immune responses in TB. These insights provide a foundation for the development of precision therapeutics that account for sex as a biological variable to improve TB outcomes.

## MATERIALS AND METHODS

### Bioethics and biosafety

All animal experimental protocols were approved by the Institutional Animal Care and Use Committee (IACUC) and the Institutional Biosafety Committee of the Center for Discovery and Innovation (CDI) at Hackensack University Medical Center, in compliance with the guidelines established by the National Research Council.

### Animal model and experimental design

Eight-week-old male and female C57BL/6J mice were obtained from the Jackson Laboratories (Bar Harbor, ME) and housed at the CDI animal research facility in sterilized filter-top cages under controlled conditions, including a 12-hour light/dark cycle, regulated humidity, and temperature. Male mice (*n* = 5) and female mice (*n* = 5) were aerosol infected with *Mtb* HN878 in BSL3 facility at Center for Discovery and Innovation of Hackensack University Medical Center. In brief, *Mtb* aerosols were generated using a Glas-Col Inhalation Exposure System (Glas-Col) with a 5 mL bacterial suspension containing approximately 3 × 10⁶ bacilli/mL in PBS with 0.04% Tween 80. Briefly, the mice were exposed to nebulized bacteria for 30 min at an inoculum dose standardized to deliver ~100 colony-forming unit (CFU) of bacteria. At 20-24 h post-aerosol infection, four mice were euthanized, and whole lungs were removed, homogenized, and plated to determine the infectious dose. Uninfected male mice (*n* = 5) and female mice (*n* = 5) were used as respective controls for the experiment. Mice were sacrificed at 30 DPI, and organs were harvested for further analysis. The right superior lobe of the lung was used to determine bacterial burden, and the postcaval lobe was stored for tissue gene expression, including *Mtb* gene expression. The right middle lobe, right inferior lobe, and left lobe were used either for histological studies, western blotting, or preparation of single cells. The flow chart showing the experimental design is presented in Fig. S1.

### Analysis of lung Mtb burden

The lung tissue was homogenized in phosphate-buffered saline with Tween (PBST), and serial dilutions of the homogenates were plated on Middlebrook 7H10 agar (Difco BD, Sparks, MD, USA) to quantify the bacterial colony-forming units (CFU). We also analyzed the *Mtb* load by qPCR analysis of *Mtb*-specific *SigA* and *Cfp10* genes, normalized to host *Hprt* and *16S* rRNA expression, respectively. Briefly, RNA was isolated from the lung tissue and reverse-transcribed from 1,000 ng of total RNA using iScript cDNA Synthesis Kit (#1708891, Bio Rad) according to the manufacturer’s protocol. The primers used for the amplification of quantitative PCR (qPCR) are listed in Table S1.

### Histological and immunofluorescence analysis of lung sections

Freshly harvested lung lobes from each mouse were fixed with 10% neutral-buffered formalin for a minimum of 48 h, then embedded in paraffin wax and sectioned for histological analyses. Hematoxylin and eosin (H&E) staining was performed on sections of both uninfected and *Mtb*-infected lungs, and the images were captured as previously described (5, 14). We used a small section of one lobe of the lung per mouse for histology. Five images (40× magnification) per section of each infected lung section were graded blindly by four investigators, and the average scores were plotted as a dot plot to analyze immune cell levels. Auramine-rhodamine (AR) staining of *Mtb*-infected lung sections was performed to detect and quantify the presence of *Mtb* burden, and the images were captured using fluorescence microscopy (15, 16). Immunofluorescence analysis (IFA) was performed on the formalin-fixed lung sections using a monoclonal anti-F4/80 antibody (1:200 dilution, NB600-404, Novus Biologicals), followed by incubation with an Alexa Fluor 555–conjugated anti-rabbit secondary antibody and 2 µg/mL Hoechst 33242 (Thermo Fisher). The stained lung sections were imaged on a Leica STEALLRIS 8 microscope using a 25 × 0.95 NA water-immersion objective. F4/80^+^ macrophage proportions (relative to the Hoechst^+^ total cells) were quantified after performing fluorescence intensity-based cell segmentation using IMARIS (Bitplane).

### Protein analysis in lung lysates

Protein lysates from lung tissue were prepared, and immunoblot analysis was conducted as previously described (5). Briefly, tissue lysates were prepared by homogenizing the lungs by using a handheld homogenizer in the presence of cell lysis buffer (9803 Cell Signaling Technology) supplemented with Pierce protease inhibitor cocktail (A32963, Thermo Fisher Scientific). The homogenate was incubated on ice for 10 min and then clarified by centrifugation at 14,000 × *g* for 15 min in a refrigerated microcentrifuge. Lysates were filtered through 0.2 µm spin columns and then removed from BSL3 facility. The supernatant was collected, and protein concentration was measured using the Pierce BCA Protein Assay Kit (#23225, Thermo Fisher Scientific). For immunoblot analysis, 30 µg of total protein from each sample was loaded onto SDS-PAGE, resolved, and transferred onto a nitrocellulose membrane. The blots were then probed with the specified primary antibodies. TNF-α–specific polyclonal antibody (#3707S, Cell signaling technology), IFN-γ–specific polyclonal antibody (#bs-0480R, Bios), IL-6–specific monoclonal antibody (#66146-1-Ig, Proteintech), pAKT (Serine 473)–specific monoclonal antibody (#4060T, Cell signaling technology), Annexin-specific polyclonal antibody (#8555s, Cell signaling technology), horseradish peroxidase (HRP)–conjugated anti-mouse immunoglobulin (#7076P2, Cell Signaling Technology), and HRP-conjugated anti-rabbit immunoglobulin (#7074P2, Cell Signaling Technology) were used as appropriate secondary antibodies to detect chemiluminescent signal using Invitrogen iBright Imaging System. Guanosine nucleotide dissociation inhibitor (GDI)–specific rabbit polyclonal antibody (#710300, Cell Signaling Technology) and No-Stain Protein Labeling Reagent (#A44717, Invitrogen) were used to normalize protein loading.

### Isolation of macrophages for RNA sequencing and qPCR analysis

Freshly harvested lung lobes (70 mg) were rinsed in PBS to remove remaining blood and dissociated to obtain single-cell suspension using the lung dissociation kit (#130-095-927, Miltenyi Biotec) in combination with the gentle MACS Octo Dissociator with heaters and C tubes (Miltenyi Biotec), following the manufacturer’s instructions. All the infected tissue samples were processed in an ABSL-3 facility at CDI RAF. The single-cell suspensions were then subjected to RBC lysis followed by MACS magnetic microbead–based cell isolation to separate and enrich the macrophage population (F4/80, #130-110-443, Miltenyi Biotec) using MultiMACS Cell24 Separator Plus system, according to the manufacturer’s instructions.

Total RNA was isolated in the lung F4/80 cells using TRIzol reagent (Invitrogen). Isolated RNA samples were treated with DNase I on column to eliminate genomic DNA contamination and further purified using the RNeasy Mini Kit (Qiagen). Purified RNA yield was assessed by NanoDrop Microvolume Spectrophotometer (Thermo Fisher Scientific). RNA isolated from lung F4/80 was sent to the Novogene sequencing facility for RNA sequencing analysis, and an aliquot of RNA was stored at −80℃ for the validation of RNA-seq data. The quality of RNA was first checked for integrity on an Agilent TapeStation; samples with RNA integrity number (RIN) >7.0 were used for subsequent processing. Total RNA was subjected to two rounds of poly(A) selection using oligo-d(T)25 magnetic beads (New England Biolabs). An Illumina-compatible RNA-seq library was prepared using NEBNext Ultra RNA-seq Library Preparation Kit. The cDNA libraries were purified using AmpureXP beads and quantified using an Agilent TapeStation and Qubit analysis. Equimolar amounts of barcoded libraries were pooled and sequenced on Illumina NovaSeq 6000 platform (Illumina, San Diego, CA), with a 2 × 150 configuration. Raw transcriptome reads were assessed for quality control (FASTQC v0.11.8) and trimmed for quality and adapter contaminant (cutadapt v 2.5). Trimmed reads were aligned to the mouse genome (GRCm38) using STAR (v2.6.1), followed by transcript abundance calculation and hit count extraction with StringTie (v2.0) and featureCounts (v1.6.4), respectively. Hit count normalization and differential gene expression group cross-comparisons were performed using DESeq2 (v1.26.0). Significant differentially expressed gene thresholds were set at FDR adjusted *P* < 0.05.

For sample size and rationale, we used three independent biological replicates per sex/experimental group (*n* = 3) for our RNA-seq experiments. This sample size reflects an exploratory, hypothesis-generating design where we prioritized depth of sequencing and stringent control of biological and technical variability (animals: same strain, age, housing, infection protocol; all samples processed in a single library preparation batch). RNA-seq is highly sensitive to detect moderate-to-large expression changes. Therefore, *n* = 3 is commonly used as a minimum for discovery studies when combined with rigorous QC and statistical modeling that borrows information across genes (e.g., DESeq2 with shrinkage estimators). To minimize false positives and increase robustness, we applied independent filtering including FDR correction. Based on the pathway analysis of differentially expressed genes, we further validated the expression of genes by qRT-PCR in independent biological replicates. We explicitly acknowledge that this sample size reduces power to detect small effect sizes, and therefore, negative findings were interpreted cautiously.

### Gene set enrichment analysis

Gene set enrichment analysis (GSEA) is a computational approach used to determine if a pre-defined gene set shows a significant consistent difference between two biological states. The genes were ranked according to the degree of differential expression in the two samples, and the predefined gene set was tested to see if they were enriched at the top or bottom of the list. Gene set enrichment analysis can include subtle expression changes. We use the local version of the GSEA analysis tool (http://www.broadinstitute.org/gsea/index.jsp). GO, KEGG, Reactome, DO, and DisGeNET data sets were used for GSEA independently.

Gene Ontology (GO) enrichment analysis of differentially expressed genes was implemented by the cluster Profiler R package, in which gene length bias was corrected. GO terms with corrected *P* value less than 0.05 were considered significantly enriched by differentially expressed genes. KEGG is a database resource for understanding high-level functions and utilities of the biological system, such as the cell, the organism, and the ecosystem, from molecular-level information, especially large-scale molecular data sets generated by genome sequencing and other high-throughput experimental technologies (http://www.genome.jp/kegg/). We used the cluster Profiler R package to test the statistical enrichment of differential expression genes in KEGG pathways. The Reactome database brings together the various reactions and biological pathways of human model species. Reactome pathways with corrected *P* values less than 0.05 were considered significantly enriched by differentially expressed genes. We used cluster Profiler software to test the statistical enrichment of differentially expressed genes in the Reactome pathway.

### Validation of RNA-seq data

RNA was reverse-transcribed from 100 ng of total RNA using iScript cDNA Synthesis Kit (#1708891, Bio Rad), and mRNA levels of *IgHg1*, *IL-1b* (Interleukin 1b), *Ctsc* (Cathepsin C), *Arg1* (Arginase), *Cacna1c* (Calcium Channel Alpha 1c), *Lamp2* (Lysosome-associated membrane protein 2), *Ulk1* (Unc-51 like autophagy activating kinase 1), *Tubb1* (Tubulin, Beta 1 Class VI), *Adra1* (Adrenergic Receptor Alpha 1), and *Col9a1* (Collagen type IX alpha 1 chain) were analyzed by qPCR using iTaq universal SYBR green super mix (#1725124, Bio Rad) and normalized to 18S levels as previously demonstrated (17). The primers used for the amplification are listed in Table S1.

### Statistical analysis

All data are expressed as mean ± SEM. Comparisons between two groups were performed using unpaired two-tailed Student’s *t*-test. *P*-values less than 0.05 were considered statistically significant. GraphPad Prism (version 10) was used for all statistical analyses.

## RESULTS

### *Mtb* load in the lungs of males was significantly higher than in females

**Fig 1:**
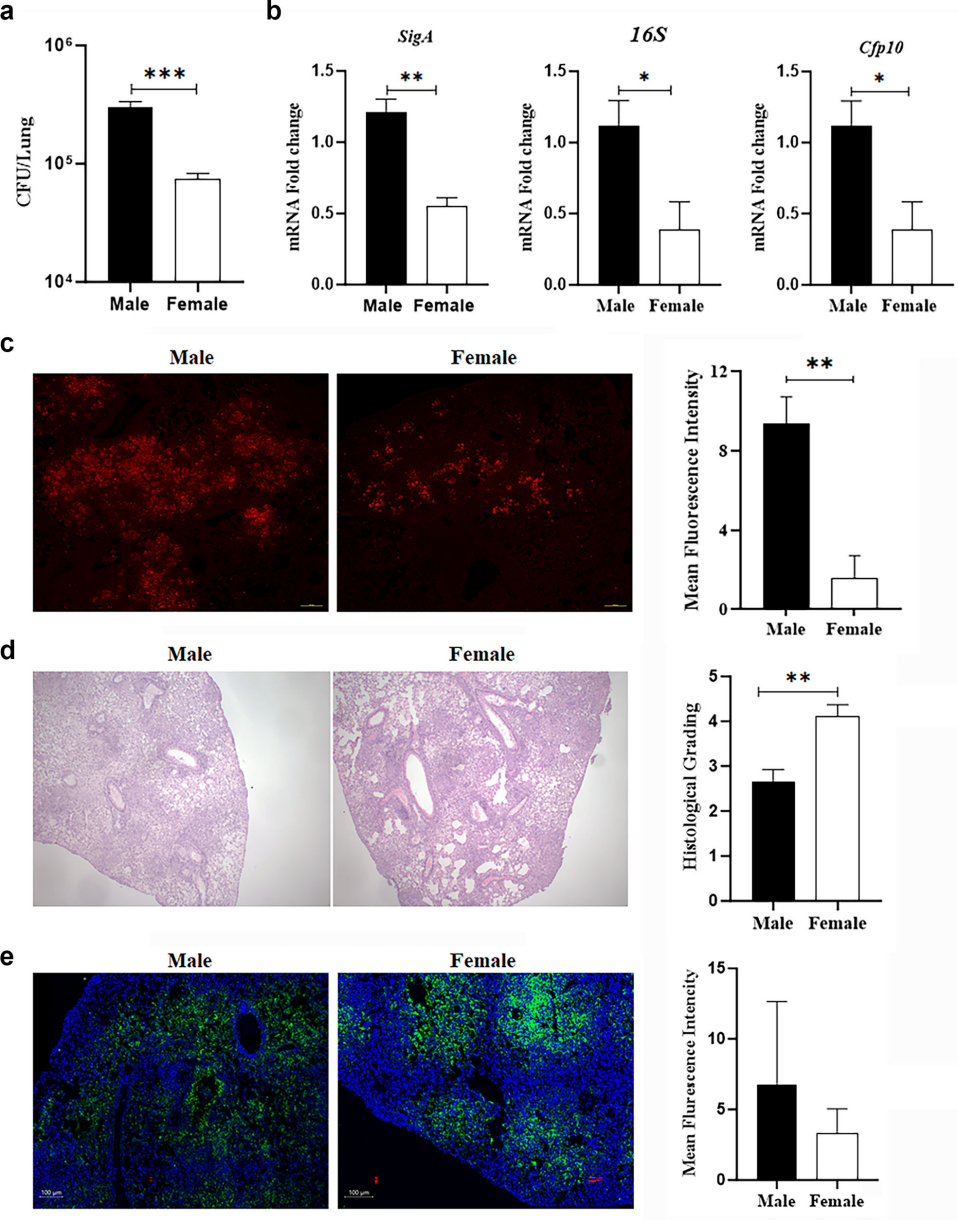
Sex-specific differences in the bacterial load, mycobacterial gene expression, histological parameters, and immune cell distribution of lung tissue during sub-acute *M. tuberculosis* infection. (**a**) Colony-forming unit (CFU) enumeration of *Mtb* in lung homogenates from male and female mice. Data are presented as mean ± SEM. (**b**) Relative expression of mycobacterial genes (*SigA*, *16S*, and *Cfp10*) in lung tissue from male and female mice, assessed by qRT-PCR. Expression levels of *SigA* and *16SrRNA* are normalized to *Hprt* and *Cfp10* were normalized to *16SrRNA*. (**c**) Auramine-rhodamine staining of lung sections highlighting *M. tuberculosis* bacilli (red) at 40x magnification. Scale bar: 100 µm. The quantification of auramine-rhodamine staining intensity was performed using ImageJ across five 40× images per mouse lung section and was shown on the right side of the image. (**d**) Hematoxylin and eosin (H&E) staining of lung sections showing granulomatous inflammation and immune cell infiltration. Images are at 10× and 40× magnification. Scale bar = 2 µm. Male and female immune cell distribution was graded in the H&E sections on a 5-point scale ranging from 0 to 5 by four independent observers included on the right side of the image. (**e**) Immunofluorescence staining of lung sections for F4/80 (green) marking macrophages. Nuclei are stained with Hoechst 33242 (blue). Scale bar: 100 µm. The quantification of fluorescent F4/80^+^ cellular proportions (relative to the total cells) per field was performed after fluorescence-based segmentation using IMARIS (Bitplane), with the bar graph shown on the right side of the image. The error bars represent the standard error of the mean. Statistical significance was calculated using a *t*-test. *P < 0.05, **P < 0.01, and ***P < 0.001 between indicated groups.

C57BL/6 mice were infected with the *Mtb* HN878 strain, and lung bacterial burden was assessed at 30 days post-infection (DPI), which represents the sub-acute stage of infection—i.e., when the acute pro-inflammatory immune response diminishes, accompanied by an increase in anti-inflammatory and resolution signaling (5, 14, 18–20). Colony-forming unit (CFU) analysis showed that lung *Mtb* burdens were significantly higher in males compared to females (Fig. 1a). We also used qPCR to analyze the levels of *Mtb* and its replication status in the lungs using HN878-*Mtb*-specific *16S*, *SigA*, and *Cfp10* genes (Fig. 1b). The 16S rRNA gene is routinely used as a molecular marker for *Mtb* identification, and its expression levels are often used as a reference or normalization standard in gene expression studies (21). The *Mtb SigA* gene is constitutively expressed and essential for regulating the transcription of numerous housekeeping genes, including those crucial for survival (22). Consistent with increased CFUs, we detected a significant increase in *16S* and *SigA* genes (*P* = 0.004 and *P* = 0.04 respectively, normalized to host *Hprt* gene expression) in the lungs of males compared to females. We also found that the expression of an *Mtb Cfp10*—a key virulence-associated factor involved in host-pathogen interactions and associated with *Mtb* replication status (23)—was significantly upregulated (*P* = 0.04, normalized to *Mtb 16S* rRNA) in the lungs of males compared to females, underscoring increased bacterial activity in male lungs at 30 DPI. Consistent with these data, microscopic analysis of auramine rhodamine-stained lung sections also demonstrated increased *Mtb* burden in the lungs in males compared to females (Fig. 1c). Together, these results revealed a higher bacterial burden and virulence-associated gene expression in male mice compared to female mice.

### Sex-dependent immune cell microenvironment in the lungs at 30 DPI

**Fig 2:**
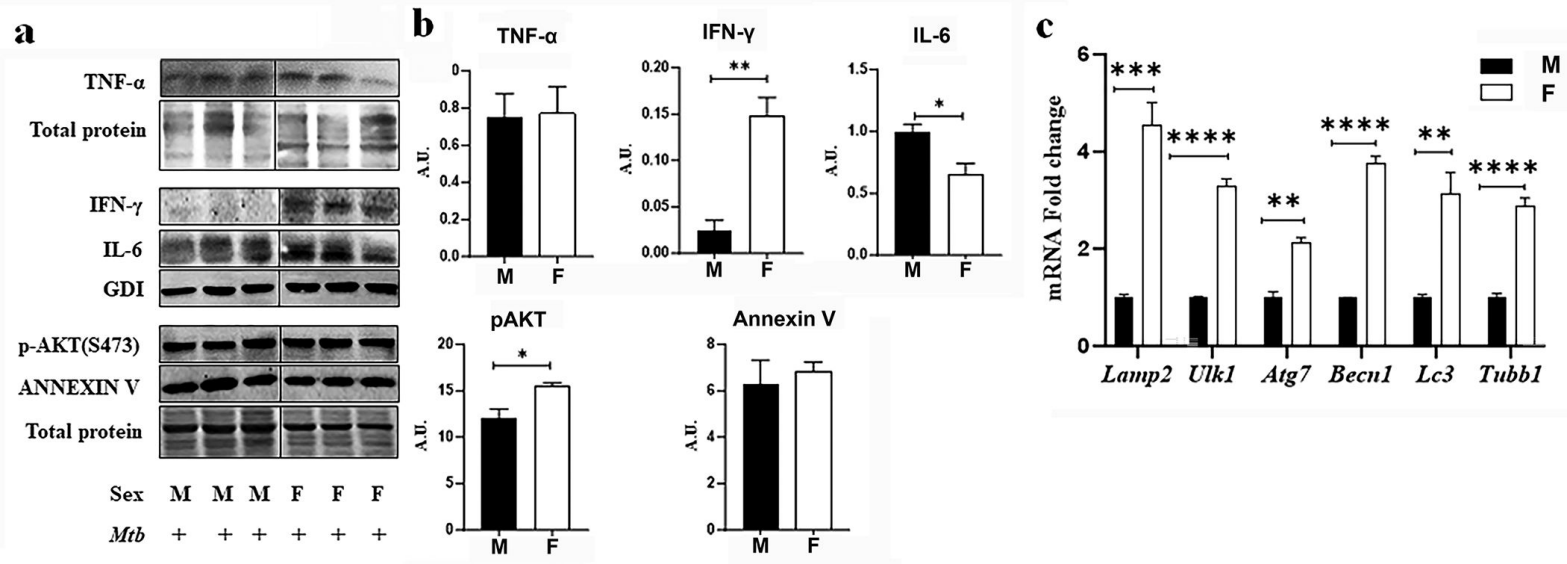
Markers of inflammation, cell survival, and autophagy exhibit sex-specific regulation in murine lungs during sub-acute *Mtb* infection. (**a**) Immunoblot analysis of TNF-α, IFN-γ, IL-6, phosphorylated AKT (p-AKT), and ANNEXIN V in lung protein lysates from male and female mice. GDI or total protein serves as the loading control. Representative blots highlight variations between sexes. (**b**) Bar graph values were derived from densitometric quantification of protein bands normalized to GDI or total protein, showing sex-related differences in inflammatory and cell survival signaling pathway. Data are presented as mean ± SEM. (**c**) qPCR analysis of autophagy-related genes (*Lamp2*, *Ulk1*, *Atg7*, *Becn1*, *Lc3*, and *Tubb1*) in lung tissue from male and female mice. Gene expression levels are normalized to 18S rRNA and presented as fold change. The error bars represent the standard error of the mean. Statistical significance was calculated using *t*-test. *P < 0.05, **P < 0.01, ***P < 0.001, and ****P < 0.0001 between indicated groups.

Alveolar macrophages are at the frontline of the immune response to *Mtb* infection because they phagocytose *Mtb* and restrict its survival and replication (8, 24) and because they release cytokines and chemokines to recruit and activate additional immune cell subsets, including neutrophils, monocytes, and lymphocytes, at the site of infection (25, 26). Our histological analysis revealed greater leukocyte infiltration (1.5-fold, *P* < 0.05) in the lungs of females compared to males (Fig. 1d). Interestingly, immunofluorescence analysis (IFA) of macrophage marker F4/80 in lung sections showed no significant difference between infected females and males (Fig. 1e), indicating that overall macrophage levels were not altered. However, western blotting analysis of lung lysates indicated that the levels of IFNγ and IL-6 were significantly altered between the sexes in the lungs of infected mice, wherein the levels of IL-6 significantly increased in males and IFNγ significantly increased in females (Fig. 2a and b). TNFα levels were similar between the sexes. Because IFNγ production can be induced by AKT activation, which promotes cell survival and proliferation (27), we used western blotting to measure AKT activation. Indeed, we observed increased levels of phosphorylated AKT in the lungs of infected females compared to males (Fig. 2a and b). Because IFNγ is known to promote autophagy, which removes damaged cellular components and is crucial for intracellular pathogen clearance and immune activation (28), we used quantitative reverse transcriptase PCR (qRT-PCR) to measure the expression of autophagy genes. Indeed, we found that lung extracts from infected females contained significantly increased mRNA levels of autophagy genes *Lamp2*, *Ulk1*, *Atg7*, *Becn1*, *LC3 (Map1LC3B)*, and *Tubb1* compared to those of infected males (Fig. 2c). Together, these data indicated that during the sub-acute stage of *Mtb* infection, the lungs of females are characterized by increased leukocyte infiltration, AKT activation, and expression of IFNγ and autophagy genes, whereas in the lungs of males, there is an induction of IL-6, a pro-inflammatory cytokine that promotes tissue damage (29).

### Sex-differentiated transcriptomic signature in lung macrophages

**Fig 3:**
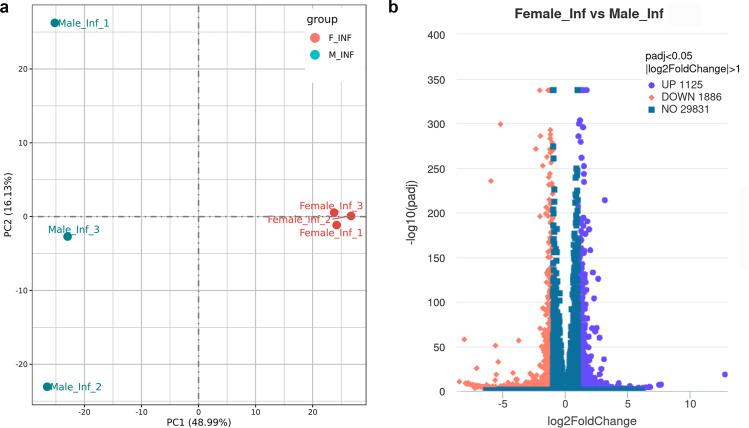
Lung macrophages isolated from *Mtb*-infected mice exhibit sex-specific transcriptomic differences. (**a**) Principal component analysis (PCA) plot showing distinct clustering of gene expression profiles in male and female *Mtb*-infected mice, indicating sex-based transcriptional differences. (**b**) Volcano plot depicting differentially expressed genes (DEGs) between male and female lung macrophages. Violet and orange dots represent significantly upregulated and downregulated genes (log2 fold change ≥ 1, adjusted *P*-value ≤ 0.05), while green dots indicate non-significant genes.

The differences in total leukocyte infiltration, inflammatory gene expression, and bacterial loads between the sexes in *Mtb*-infected mice at 30 DPI suggested that despite similar macrophage levels, there may be sex-specific differences in macrophage transcriptomes. To examine the role of sex in macrophage transcriptome reprogramming, we performed RNA sequencing (RNA-seq) analysis of lung macrophages isolated from *Mtb*-infected mice at 30 DPI. The RNA-seq analysis revealed several significant differences in lung macrophage gene expression between the sexes (Fig. 3a). A total of 3,011 differentially expressed genes (DEGs) were identified, with 1,886 genes significantly downregulated and 1,125 genes significantly upregulated in macrophages from females compared to males (Fig. 3b). Notably, the most upregulated genes (Table 1) in females compared to males included those involved in tissue repair (*Col9a1*, *Krt5*), anti-inflammatory signaling (*Vsig4* [30]), and phagocytosis (*Slc22a12* [31]), as well as sex chromosome-linked genes. A particularly prominent example was *Xist*, a long non-coding RNA (lncRNA) crucial for X-chromosome inactivation, which showed a log₂ fold change of 12.76 (Table 1). Increasing evidence suggests that lncRNAs, such as *Xist*, play essential roles in regulating macrophage function and modulating disease progression (32).

**TABLE 1:** Significantly downregulated and upregulated genes in female macrophages compared to male at 30 DPI

Gene ID	log2 FC	*P* adj
*Ighg1*	−8.5	2.50E-11
*Ighg2b*	−5.4	1.80E-33
*Ighg2c*	−5.2	1.10E-299
*Ighg3*	−5.6	2.10E-51
*Kdm5d*	−8.1	3.43E-58
*Uty*	−7.8	2.44E-09
*Uba1y*	−7.9	9.86E-09
*Xist*	12.8	4.89E-19
*Col9a1*	7.6	9.02E-08
*Krt5*	7.5	1.69E-07
*Slc22a12*	6.8	3.67E-05
*Vsig4*	6.7	2.31E-05
*Gm34921*	6.7	5.16E-05
*Hhipl2*	6.5	0.000120
*Gm13063*	6.4	0.000249
*Gm13748*	6.3	0.000328
*Gm37986*	6.2	0.000584

In males, as expected, the expression of Y-chromosome-related genes, such as *Kdm5d*, *Uba1y*, and *Uty*, was significantly increased in macrophages from male mice compared to female mice. Furthermore, a significant proportion of upregulated genes in males was associated with immunoglobulin heavy chains. Among these, *Ighg1*, which encodes the constant region of the IgG1 immunoglobulin heavy chain, was the most significantly upregulated gene, exhibiting a log_2_ fold change of 8.48 (Table 1). In addition to the marked upregulation of *Ighg1*, we observed significant sex-dependent differences in the transcription of immunoglobulin kappa variable (*Igkv*) and heavy variable (*Ighv*) genes in lung macrophages from HN878-infected mice (Table 2). For example, the log_2_ fold increases in the levels of *Ighv1-50*, *Igkv6-23*, *Igkv6-20*, *Ighv1-80*, *Ighv2-2*, *Igkv3-12*, *Igkv4-59*, and *Ighv1-75* were greater than 7, and these genes are among the top 15 significantly altered genes in macrophages between the sexes at 30 DPI. Our findings reveal a sex-specific divergence in macrophage transcriptomic remodeling during the post-acute stage of *Mtb* infection, characterized by elevated immunoglobulin gene expression in males and increased expression of anti-inflammatory and tissue repair genes in females, suggesting that this differential regulation may critically influence macrophage immune functions and the intracellular replication dynamics of *Mtb* in the lung.

**TABLE 2:** RNA sequencing analysis showed significant upregulation of immunoglobulin kappa chain variables, in male macrophages when compared to females at 30 DPI, and immunoglobulin heavy chain variables, in male macrophages when compared to females

Gene ID	log2 FC	*P* adj
Immunoglobulin kappa chain variables		
*Igkv17-127*	−4.0	1.20E-13
*Igkv10-96*	−5.6	1.00E-12
*Igkv6-23*	−8.0	4.20E-09
*Igkv6-20*	−8.0	8.70E-09
*Igkv6-17*	−3.9	4.40E-08
*Igkv1-135*	−4.9	2.20E-07
*Igkv4-59*	−7.3	5.90E-07
*Igkv14-111*	−4.5	6.80E-07
*Igkv3-12*	−7.3	7.80E-07
*Igkv1-117*	−2.9	2.40E-06
*Igkv16-104*	−4.1	1.10E-05
*Igkv6-32*	−6.8	4.20E-05
*Igkv6-15*	−3.0	5.70E-05
*Igkv3-2*	−3.75	0.000138
*Igkv12-44*	−2.78	0.000491
*Igkv4-57*	−4.05	0.000752
*Igkv4-72*	−4.72	0.000758
*Igkv4-57-1*	−3.71	0.000888
*Igkv4-79*	−5.94	0.001675
*Igkv19-93*	−3.55	0.001992
*Igkv5-43*	−3.47	0.004505
*Igkv3-7*	−5.57	0.007012
*Igkv8-30*	−4.53	0.009474
*Igkv4-86*	−3.63	0.012649
*Igkv4-53*	−5.35	0.016377
*Igkv10-94*	−4.20	0.016925
*Igkv9-120*	−2.64	0.029689
*Igkv4-68*	−2.59	0.043414
*Igkv8-28*	−3.19	0.044394
*Igkv4-81*	−5.01	0.047051
*Igkv11-125*	−4.98	0.048906
Immunoglobulin heavy chain variables		
*Ighv1-82*	−4.59	4.90E-10
*Ighv1-50*	−7.98	4.20E-09
*Ighv1-80*	−7.76	1.50E-08
*Ighv9-3*	−4.20	1.90E-08
*Ighv1-64*	−6.27	1.40E-07
*Ighv2-2*	−7.34	3.20E-07
*Ighv1-18*	−6.98	3.40E-06
*Ighv1-39*	−2.91	6.10E-05
*Ighv14-4*	−6.44	0.000101
*Ighv3-5*	−6.58	0.000125
*Ighv3-6*	−2.67	0.001162
*Ighv1-72*	−3.95	0.001350
*Ighv1-74*	−5.92	0.001610
*Ighv1-75*	−7.24	0.001699
*Ighv1-59*	−5.88	0.001876
*Ighv1-55*	−2.48	0.002176
*Ighv8-8*	−5.29	0.002564
*Ighv2-3*	−3.47	0.004789
*Ighv1-81*	−5.57	0.007328
*Ighv5-15*	−5.51	0.010631
*Ighv10-3*	−5.38	0.014882
*Ighv2-9*	−4.10	0.023002
*Ighv1-53*	−2.70	0.026476
*Ighv6-6*	−3.63	0.039465
*Ighv1-20*	−5.27	0.043829
*Ighv5-9-1*	−3.42	0.048546

**Fig 4:**
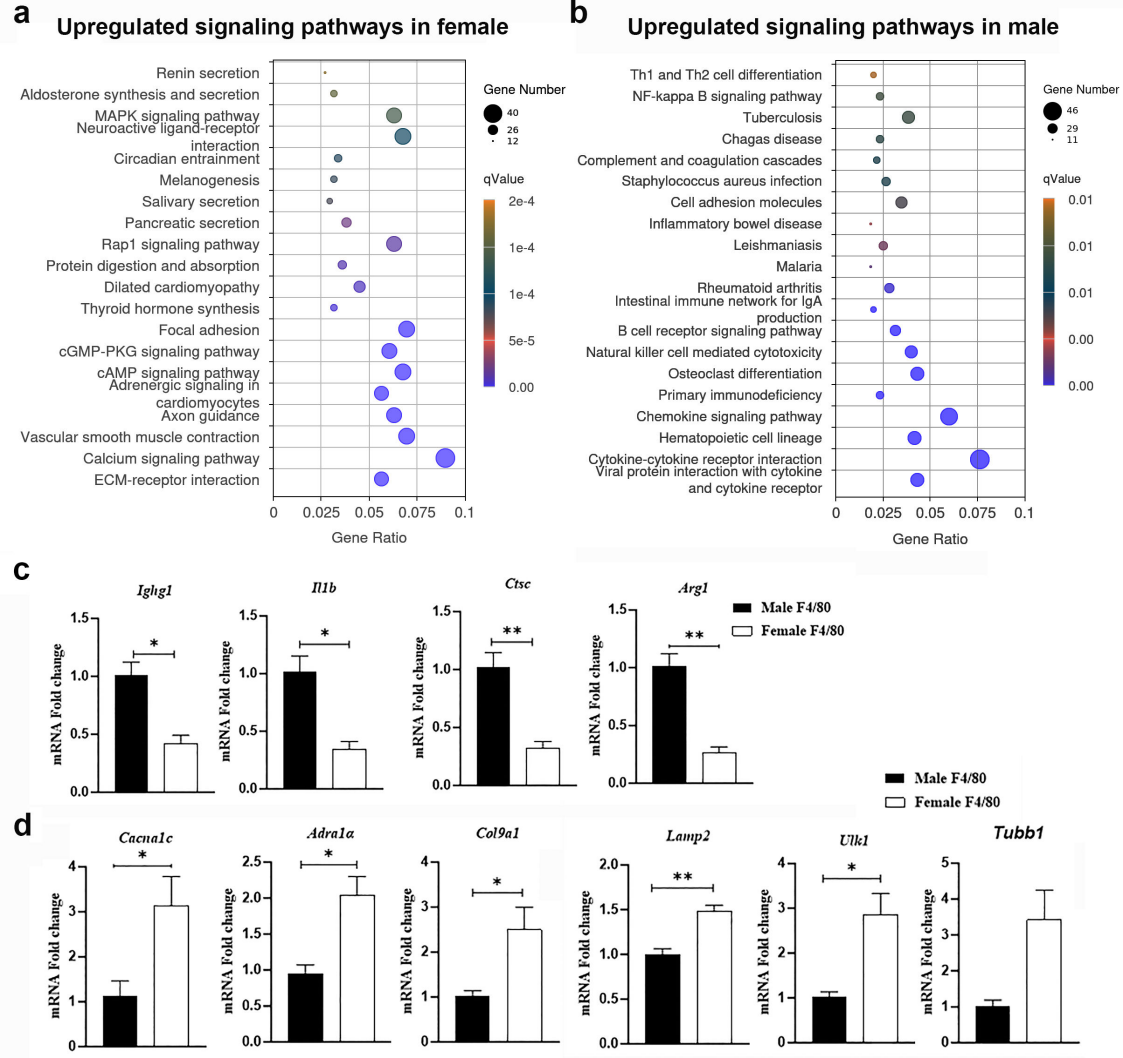
Differentially expressed genes in lung macrophages isolated from *Mtb*-infected male and female. (**a**) KEGG pathway enrichment analysis showing upregulated signaling pathways enriched in the lung macrophages from the female mice in response to *Mtb* infection. (**b**) KEGG pathway enrichment analysis of upregulated genes in lung macrophages from the male mice, illustrating pathways that are preferentially activated in the male mice in response to infection. (**c**) qPCR validation of RNA-seq data in lung macrophages from *Mtb*-infected mice. Quantitative PCR (qPCR) analysis confirming the differential expression of genes upregulated in male macrophages, including *Ighg1*, *Il1b*, *Ctsc*, and *Arg1*, showing higher expression levels in males compared to females identified by RNA sequencing. (**d**) Genes upregulated in female macrophages, including *Cacna1c*, *Lamp2*, *Ulk1*, *Tubb1*, *Adra1a,* and *Col9,* exhibiting higher expression in females. Gene expression levels are normalized to 18S rRNA and presented as fold change. Data are shown as mean ± SEM. Statistical significance was determined using a *t*-test between infected male and female mice. *P < 0.05, **P < 0.01 between indicated groups.

To gain further insights into lung macrophage transcriptional processes regulated by sex during acute TB, we analyzed macrophage transcriptomes using gene set enrichment analysis (GSEA), Gene Ontology (GO), KEGG, and Reactome pathway analysis tools. We observed significant sex-specific differences in the molecular functions, cellular components, biological processes, and pathways, including metabolic and regulatory networks (Fig. 4a, b, and 5a through d). Key findings are presented below.

**Fig 5:**
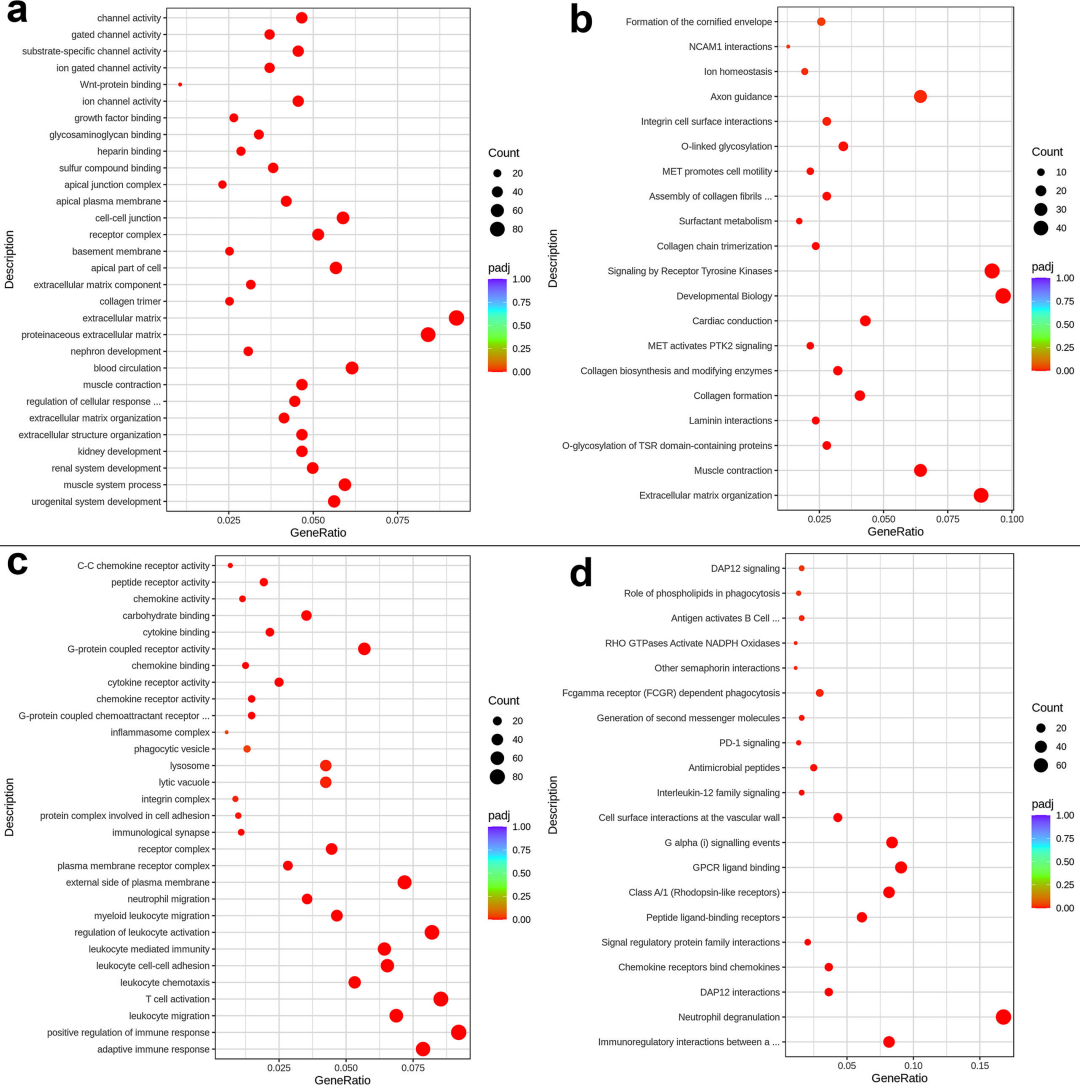
GO and REACTOME pathway analysis showing upregulated pathways in females (**a and b**) and in males (**c and d**).

#### Elevated tissue remodeling and wound healing process signaling in lung macrophages in female mice

Pathway analysis showed significantly upregulated genes involved in extracellular matrix (ECM) organization and ECM receptor interaction pathways in female macrophages compared to males (Table S2a and b). Macrophages influence the ECM by producing enzymes that degrade its components, secreting ECM proteins, and responding to its signals. These activities are critical for tissue remodeling and wound healing (33). Pathway analysis also showed greater levels of cAMP signaling pathway genes, which promote wound healing, in female macrophages compared to males (34) (Table S2c).

#### Elevated calcium signaling and autophagy gene expression in lung macrophages in female mice

Pathway analysis revealed significant upregulation of calcium signaling and ion channel activity genes in female macrophages compared to males (Table S3a and b). This may be significant because calcium signaling supports phagosome formation, chemotactic responses, cytokine secretion, and autophagy (35, 36). Pathway analysis also showed elevated cGMP-PKG pathway-related genes, which regulate Ca²^+^, mitochondrial function, and autophagy (37), in female macrophages (Table S3c).

#### Genes associated with lipophagy and lipid efflux signaling were significantly upregulated in female lung macrophages

Female macrophages showed upregulated adrenergic signaling (Table 3) and genes related to lipolysis, lipid degradation, and lipid efflux, including *AdipoQ, Plin4, Lamp3*, and ATP-binding cassette genes (Table 3). This may correspond to reduced foam cell formation and therefore reduced bacterial persistence (38) in *Mtb*-infected macrophages in females (13).

**TABLE 3:** RNA seq data revealed elevated adrenergic signaling and lipophagy and lipid efflux signaling in female macrophages at 30 DPI

Gene ID	Log2 FC	*P* adj
Adrenergic signaling		
*Atp1a2*	1.28	4.12E-114
*Plcb4*	1.32	2.84E-82
*Cacna1d*	1.27	1.05E-60
*Cacna1c*	1.40	7.05E-40
*Rapgef3*	1.05	5.45E-38
*Adra1a*	1.31	8.40E-25
*Atp1b1*	1.40	2.25E-22
*Cacna2d1*	1.10	2.25E-22
*Tpm2*	1.22	1.25E-21
*Actc1*	1.68	8.07E-20
*Adcy9*	1.02	6.30E-16
*Adcy8*	1.03	2.00E-15
*Adcy5*	1.43	2.58E-09
*Agtr1a*	1.25	1.03E-07
*Popdc2*	1.19	2.94E-07
*Cacng7*	1.60	5.80E-06
*Popdc3*	1.44	2.11E-05
*Adra1b*	1.71	3.51E-05
*Gm7308*	4.10	0.000685
*Gm35161*	6.07	0.001399
*Adra1d*	1.76	0.005558
*Atp2a1*	1.33	0.009731
*Camk2a*	1.02	0.013918
*Scn5a*	1.36	0.017913
*Cacna2d3*	1.03	0.034248
Lipophagy and lipid efflux signaling		
*Adipoq*	3.79	0.0178
*Plin4*	2.16	0.0000
*Lamp3*	1.39	0.0000
*Abcg2*	1.14	0.0000

#### Female lung macrophages exhibited elevated cell survival and proliferation signaling compared to males

Pathway analysis revealed significant increases in pathways promoting macrophage survival, inflammation resolution, and tissue repair, such as WNT and PI3K-AKT signaling (39), in female macrophages (Table S4a and b).

#### Upregulated adaptive response signaling in male macrophages

Pathway analysis identified significant upregulation of 72 genes associated with adaptive response signaling in male macrophages (Table S5a).

#### Upregulation of immune response signaling pathways in male macrophages

Male macrophages exhibited significantly higher expression of genes associated with immune response regulation, leukocyte migration, and T cell activation, represented by 83, 62, and 77 genes, respectively (Fig. 4b; Table S5b through d). Cytokine-cytokine receptor interaction and chemokine signaling pathways were also notably upregulated (Table S6a and b). Key upregulated genes included *Il1b*, *Il12b*, *Ccl8*, *Ccl3*, *Ccl6*, *Ccl5*, *Cxcr3*, *Cxcr2*, *Il23a*, *Cxcl3*, *Cxcr5*, *Ccl1*, *Ccr6*, *Cxcl16*, and *Ccr2* (Table S6c). Furthermore, DAP12 signaling was markedly elevated in male macrophages, a pathway known to promote pro-inflammatory cytokine production (40). DAP12 overexpression drives macrophage fusion and the formation of multinucleated giant cells, a hallmark of granulomatous inflammation (41). Giant cells act as antigen-presenting cells (APCs), displaying *Mtb* antigens to the immune system, particularly CD4^+^ T cells, within granulomas (42). These findings underscore the enhanced immune and inflammatory responses, as well as adaptive functions, in male macrophages compared to females at 30 DPI.

### Validation of RNA-seq results by quantitative reverse transcriptase PCR (qRT-PCR)

To validate key RNA-seq findings, we re-isolated RNA from lung macrophages of infected mice and performed qRT-PCR analysis of selected genes of interest shown by RNA-seq as significantly altered between the sexes during infection, including *Ighg1*, *Il1b*, *Ctsc*, *Cacna1c*, *Lamp2*, *Ulk1*, *Tubb1*, *Adra1a*, and *Col9a1* (Fig. 4c and d). The qRT-PCR data confirmed the upregulation of *Ighg1*, *Il1b*, and *Ctsc* in male macrophages, reinforcing the observed male-biased enhancement of immune response and inflammatory signaling pathways. *Ighg1* encodes a protein involved in adaptive immune responses, functioning as part of the IgG1 antibody, which is crucial for neutralizing pathogens and activating complement-dependent cytotoxicity (43). In contrast, *Il1b* and *Ctsc* promote pro-inflammatory signaling (44, 45). Female macrophages exhibited increased expression of genes, such as *Cacna1c*, *Lamp2*, *Ulk1*, *Tubb1*, *Adra1a*, and *Col9a1*, which are known to be involved in cellular remodeling, autophagy, and essential macrophage functions (Fig. 4d).

Finally, we used qRT-PCR to compare the expression of several genes involved in autophagy/lysosomal function and Akt signaling in macrophages derived from infected lungs with that in extracts of whole infected lungs. Consistent with RNA-seq and qRT-PCR findings in macrophages, qRT-PCR analysis of whole lung tissue revealed increased expression of *Lamp2*, *Ulk1*, *Atg7*, *Becn1*, *Lc3* (*Map1Lc3b*), *Tubb1*, *Cacna1c*, *Adra1α*, and *Col9a1* genes in females (Fig. 2c and 4d). These results are consistent with increased phagocytic activity in female macrophages relative to male macrophages, which likely contributes to more effective bacterial containment and reduced *Mtb* burden. Furthermore, increased Akt signaling—critical for cell survival and autophagy—was observed in female infected lung tissue, as it had been in female lung macrophages, correlating with decreased tissue damage and cell death (Fig. 2a and b).

## DISCUSSION

Our histological and CFU analyses revealed that despite comparable levels of F4/80^+^ macrophages in male and female lungs during sub-acute *Mtb* infection (30 DPI), male lungs harbored a significantly higher *Mtb* burden. This suggests that macrophages in males may be less efficient at controlling bacterial replication. To explore the underlying mechanisms, we performed transcriptomic profiling and qRT-PCR validation of selected genes, which provided the first evidence of sex-specific transcriptional reprogramming in lung macrophages during this infection stage. Although macrophage abundance was similar between the sexes during infection, their transcriptional profiles diverged substantially, suggesting intrinsic functional differences (transcriptional analysis in lung macrophages from uninfected mice was not carried out as their abundance is relatively low). Gene set enrichment analysis showed that female macrophages upregulated genes involved in phagocytosis, tissue remodeling, wound healing, autophagy/lipophagy, and cell survival. In contrast, genes more highly expressed in male macrophages were enriched for adaptive immune responses and immunoglobulins, including sex-specific regulation of immunoglobulin heavy and light chain variable genes. This emerging area of macrophage biology points toward a potential immunomodulatory role that may influence infection outcomes in sex-dependent fashion (46, 47).

In the context of immune signaling, female lungs demonstrated higher lymphocyte aggregation and elevated IFNγ expression, while males exhibited increased IL-6 levels. IFNγ, primarily secreted by T and NK cells, enhances macrophage-mediated antibacterial mechanisms, such as autophagy and phagolysosomal maturation (28, 48). IL-6, although a component of adaptive immunity, has been associated with heightened inflammation and impaired *Mtb* clearance when chronically elevated (49, 50). Consistent with this, we observed enhanced AKT signaling—a pathway that synergizes with IFNγ to support autophagy and intracellular bacterial killing—in females. Further pathway analysis revealed increased cytokine receptor activity and chemokine signaling in male macrophages—processes that can exacerbate inflammation and promote immune dysregulation, potentially favoring extracellular *Mtb* survival (51, 52). Conversely, female macrophages upregulated WNT and PI3K-AKT pathways, which are associated with macrophage survival, M2 polarization, and tissue repair (39, 53). Additionally, higher intracellular cAMP levels detected in females support a wound-healing, anti-inflammatory macrophage phenotype (34, 54). Finally, female macrophages activated lipophagy and cholesterol efflux pathways, which can protect against foam cell formation and promote bacterial clearance (55–60). Together, these findings support the hypothesis that sex-specific differences in AKT-IFNγ, WNT, and PI3K-AKT and autophagic/lysosomal signaling, as well as in lipid metabolism, contribute to more efficient bacterial control in females by enhancing macrophage phagocytic and lysosomal functions and reducing tissue damage.

An intriguing and underexplored aspect of our findings is the potential role of macrophages in humoral immunity. While the TB pathogenesis field has traditionally focused on Th1-driven cellular immunity, there is growing recognition of antibody-mediated mechanisms (61, 62). In our study, male macrophages showed upregulation of *Ighg1*, a gene encoding the Fc portion of IgG1, which is involved in antibody-dependent cellular cytotoxicity and complement activation (NCBI Gene ID: 3500). Although the function of immunoglobulin expression in macrophages is not fully understood, emerging studies suggest potential sex-specific regulation of macrophage responses, and these cells may actively participate in shaping humoral responses (63, 64). This warrants further investigation into the role of immunoglobulin expression in macrophage polarization and antimicrobial defense.

### Limitation

We recognize that *n* = 3 limits sensitivity for detecting small transcriptomic changes and increases false-negative risk. To address this, we used conservative statistical thresholds, reported effect sizes, and validated all priority candidates independently. Future confirmatory studies will include larger cohorts to quantify smaller or subtler transcriptional differences.

In conclusion**,** our findings demonstrate that sex is a critical determinant of macrophage function during sub-acute *Mtb* infection. While male macrophages displayed greater adaptive immune gene expression and inflammatory signaling, which correlated with higher bacterial loads, female macrophages favored autophagy, lipid dynamics, survival signaling, and tissue repair—all of which are conducive to effective *Mtb* clearance. These data reveal a previously unappreciated layer of immune regulation based on sex and underscore the necessity of incorporating sex as a biological variable in TB research. Altogether, our study underscores that understanding the molecular and metabolic basis of these sex-specific immune responses is essential for designing targeted interventions. Future research is necessary to leverage these differences to develop sex-informed therapies that enhance protective macrophage functions and limit immunopathology in TB and other infectious diseases.

## SUPPLEMENTAL MATERIAL

Supplemental figures and tables spectrum.01790-25-s0001.docx Figure S1 and Tables S1 to S6.

ASM does not own the copyrights to Supplemental Material that may be linked to, or accessed through, an article. The authors have granted ASM a non-exclusive, world-wide license to publish the Supplemental Material files. Please contact the corresponding author directly for reuse.
