# Supplementary material for: Sex-specific transcriptional regulation in lung macrophages during sub-acute Mycobacterium tuberculosis infection
Source: Microbiol Spectr. 2025 Nov 12;13(12):e01790-25. doi: 10.1128/spectrum.01790-25 (PMC12671073; doi:10.1128/spectrum.01790-25)

**Supplemental Figure 1.** Experimental design and sample distribution for control and *Mtb*–infected mice. Flowchart summarizing the experimental workflow. A total of 5 male and 5 female mice were included in each group (control and infected). At 30 days post-infection, lungs were harvested. For infected group, the left lung lobe was used for macrophage isolation (n = 3/sex). The right lung lobe was divided into sections: one portion for CFU (N=5/sex) one portion for histology (n = 5/sex), one for protein analysis (n = 3/sex), and one for RNA isolation (n = 3/sex). For control group, the left lung lobe was used for macrophage isolation (n=3/sex). Right ling lobe were divided into different sections: one section for histology (n=5/sex), one section for protein (n=3/sex) and another section for RNA (n=3/sex).

**
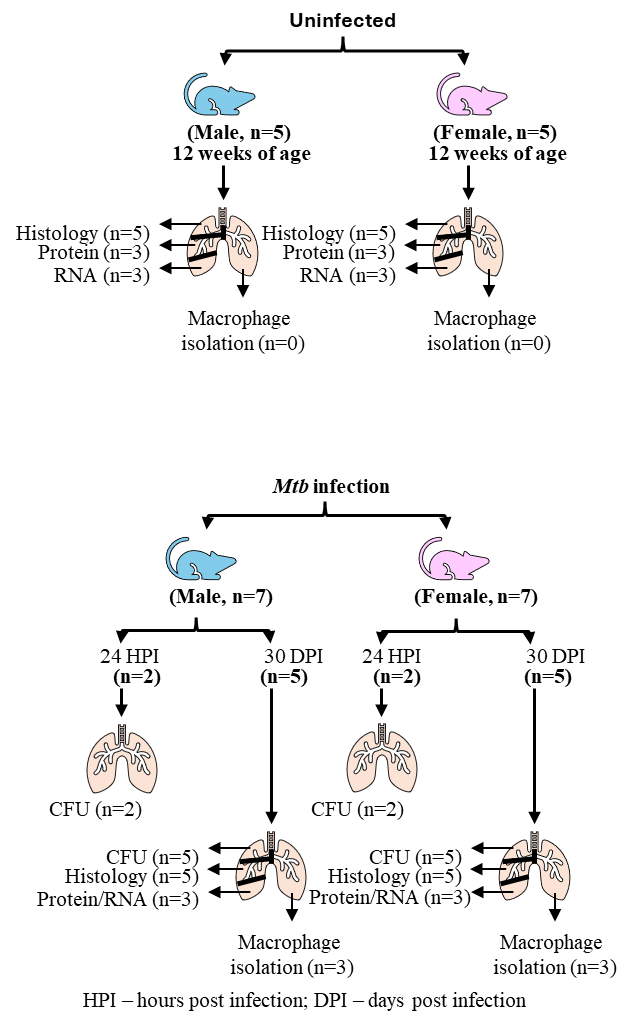
**

**Supplemental Table 1.** Primer sequences used for real-time qPCR.

| **Primer** | **Forward** | **Reverse** |
| --- | --- | --- |
| *IgHg1* | TGGGCAGCCAGCGGAGAACT | GGCTCTTCTCAGTATGGTGG |
| *Ctsc* | CTGCTTTCCCTACACAGCCA | ACGGAGGCAATTCTCCCTTG |
| *Cacna1c* | CGTTCTCATCCTGCTCAACA | TATGCTCCCAATGACGATGA |
| *Il1b* | TGGACCTTCCAGGATGAGGACA | GTTCATCTCGGAGCCTGTAGTG |
| *Adra1a* | TGGCTGCCATTCTTCCTCGTGA | TTCTTGAACTCCTGGCTGGAGC |
| *Col9a1* | ATGGCCTGGGCTGCCTGG | CCGGAACTCCAGGAGGC |
| *Arg1* | TCACCTGAGCTTTGATGTCG | CTGAAAGGAGCCCTGTCTTG |
| *Lamp2* | GAGCAGGTGCTTTCTGTGTCTAG | GCCTGAAAGACCAGCACCAACT |
| *Ulk1* | GCAGCAAAGACTCCTGTGACAC | CCACTACACAGCAGGCTATCAG |
| *Tubb1* | GGAGTCCTCTTTCAGCCTGACA | CTCCCTCCTTACCACATCCATG |
| *Atg7* | CCTGTGAGCTTGGATCAAAGGC | GAGCAAGGAGACCAGAACAGTG |
| *Becn1* | CAGCCTCTGAAACTGGACACGA | CTCTCCTGAGTTAGCCTCTTCC |
| *LC3* | GTCCTGGACAAGACCAAGTTCC | CCATTCACCAGGAGGAAGAAGG |
| *HPRT* | GTTGGATCAAGGCCAGACTTTGTT | GAGGGTAGGCTGGCCTATAGGCT |
| *18SrRNA* | CGGAAAATAGCCTTCGCCATCAC | ATCACTCGCTCCACCTCATCCT |
| *SigA* | CTCGACGCTGAACCAGACCT | AGGTCTTCGTGGCTTCGTC |
| *16SrRNA* | CCGCGGCCTATCAGCTTGTTGGT | GTAGTTGGCCGGTGCTTCTTCTCC |
| *CFP10* | GCAGGAGGCAGGTAATTTCG | CCTGGTCGATCTGGGTTTTC |

**Supplemental Table 2.** Pathway analysis revealed increased activation of tissue remodeling and wound healing processes in female macrophages at 30 DPI. Specifically, gene expression related to (a) ECM organization, (b) ECM receptor interaction, and (c) cAMP signaling pathways was significantly upregulated in female macrophages compared to males.


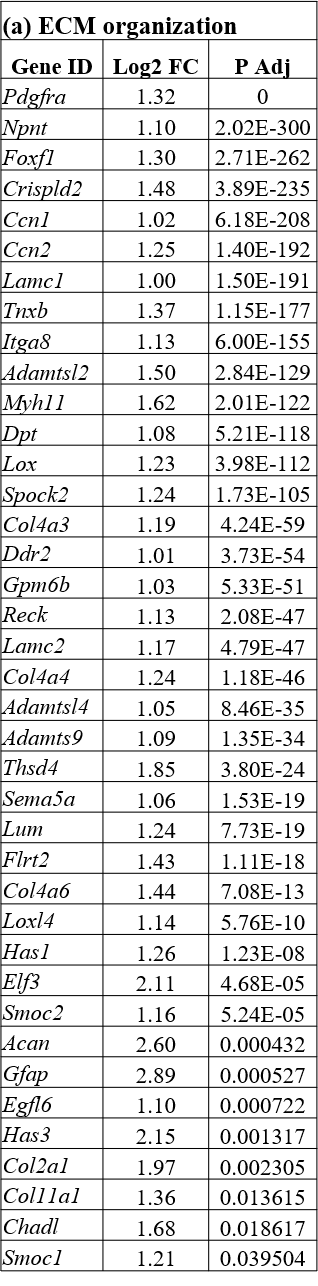

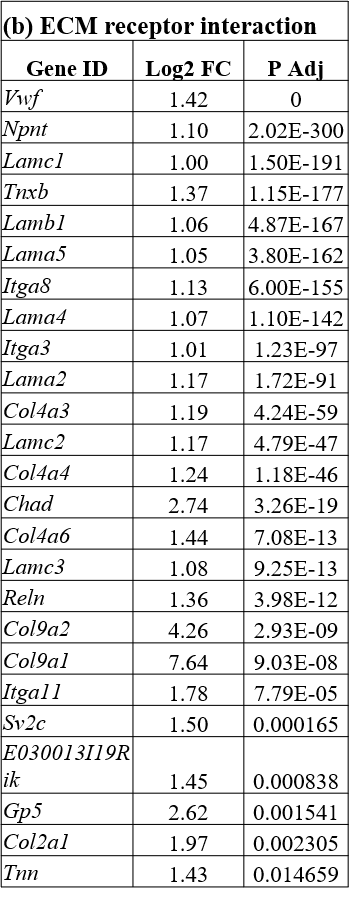

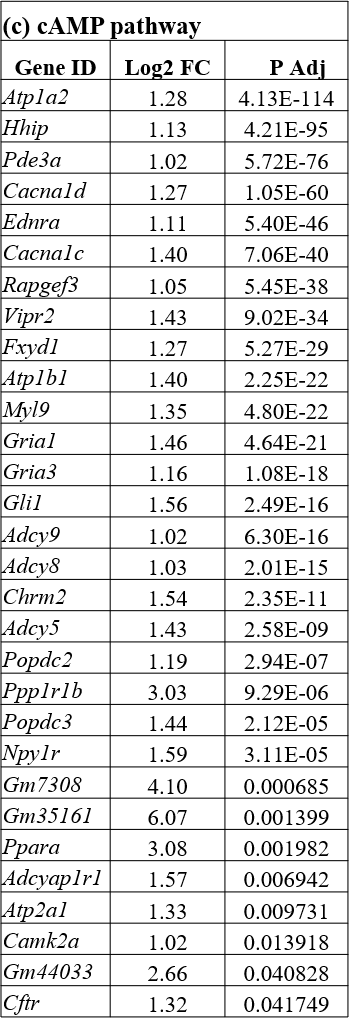


**Supplemental Table 3.** At 30 DPI, female macrophages exhibited upregulation of phagocytosis and autophagic signaling, with particularly increased (a) calcium signaling, (b) ion channel activity, and (c) cGMP-PKG pathway.


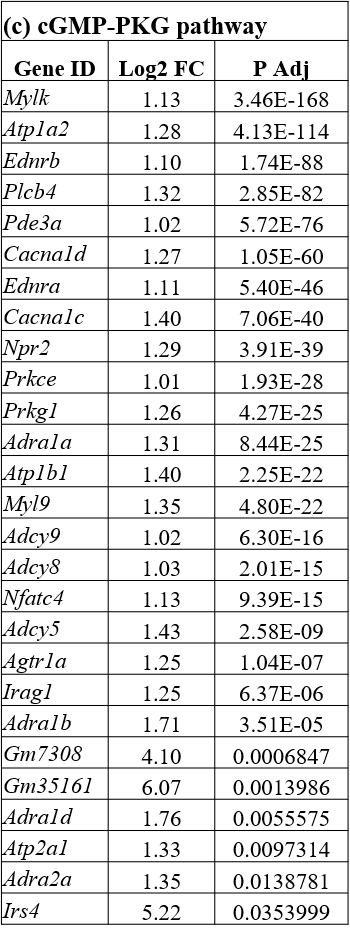

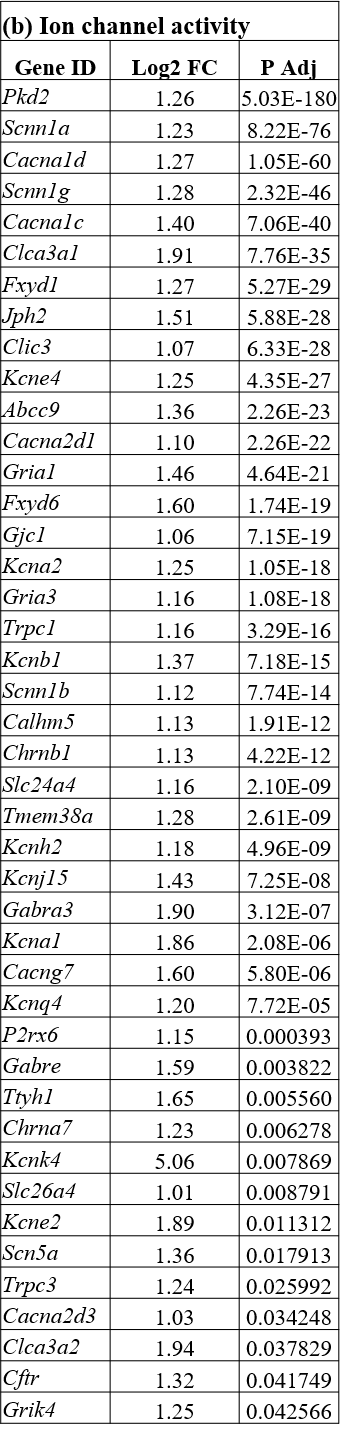

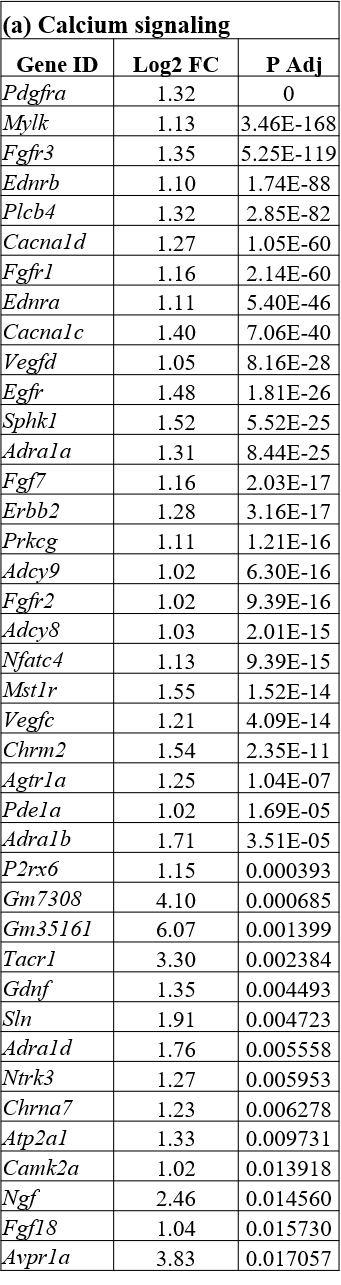


**Supplemental Table 4.** RNA sequencing analysis showed elevated cell survival and proliferation signaling, indicated by elevated expression of genes associated with (a) WNT and (b) PI3-AKT signaling.


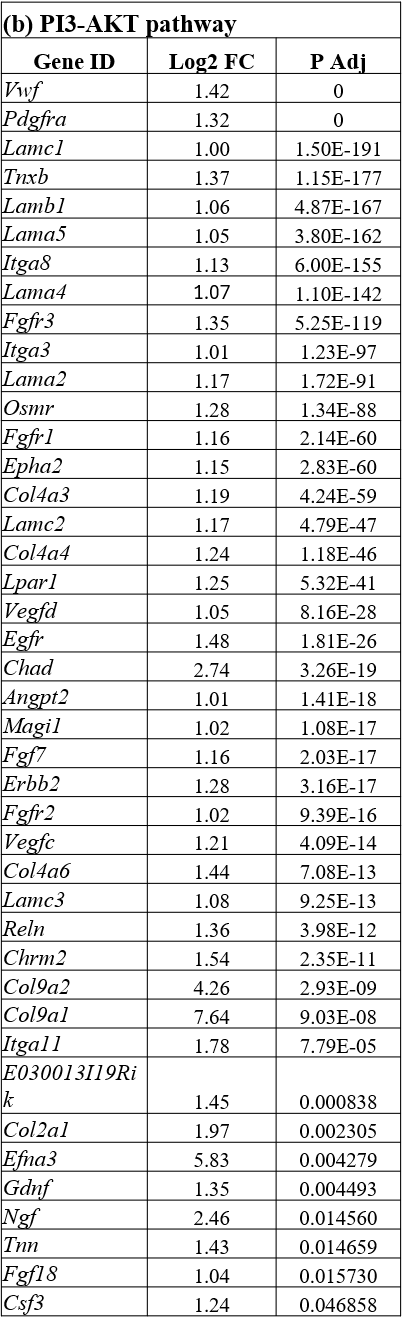

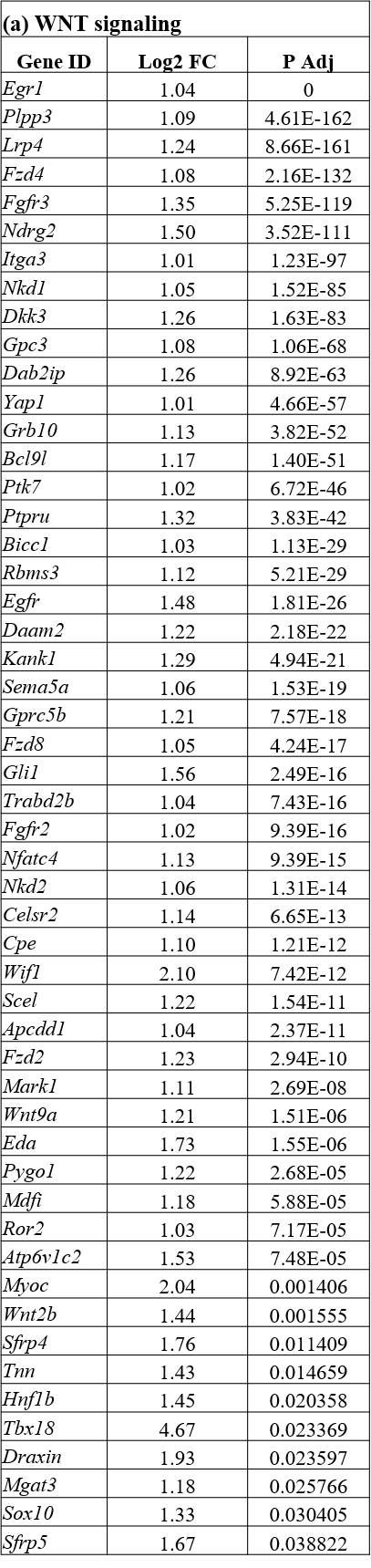


**Supplemental Table 5.** Pathway analysis showed upregulated (a) adaptive response signaling, (b) immune response regulation, (c) leukocyte migration, and (d) T-cell activation in male macrophages.


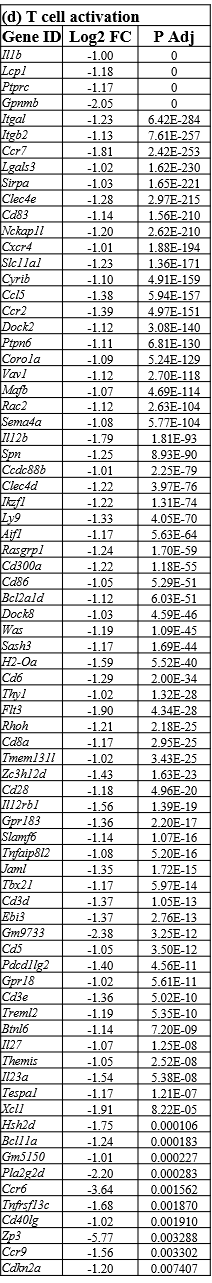

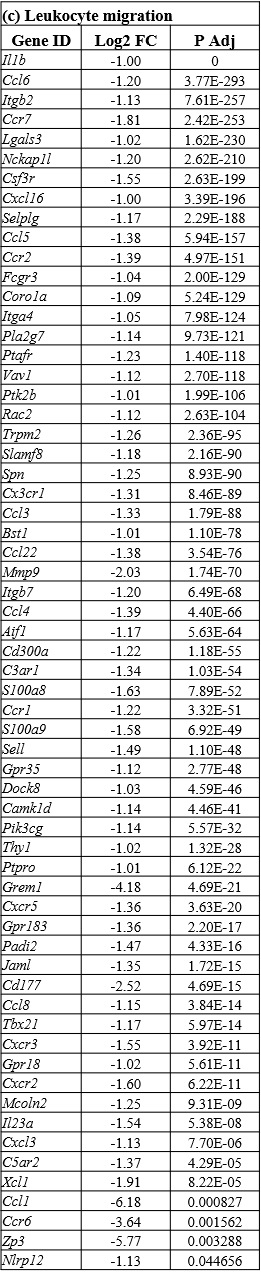

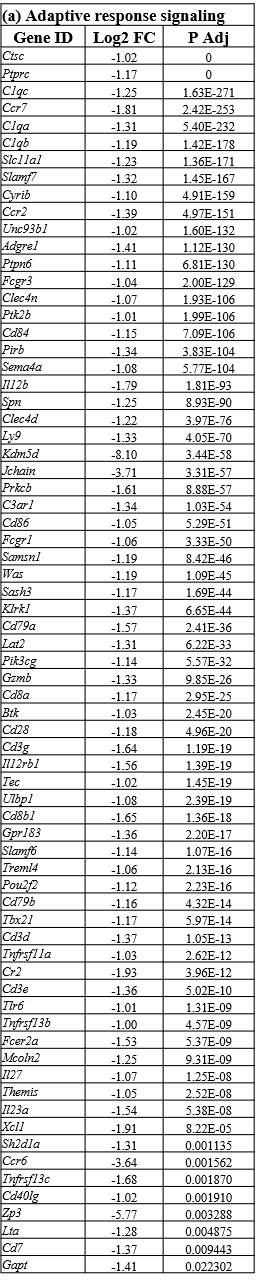

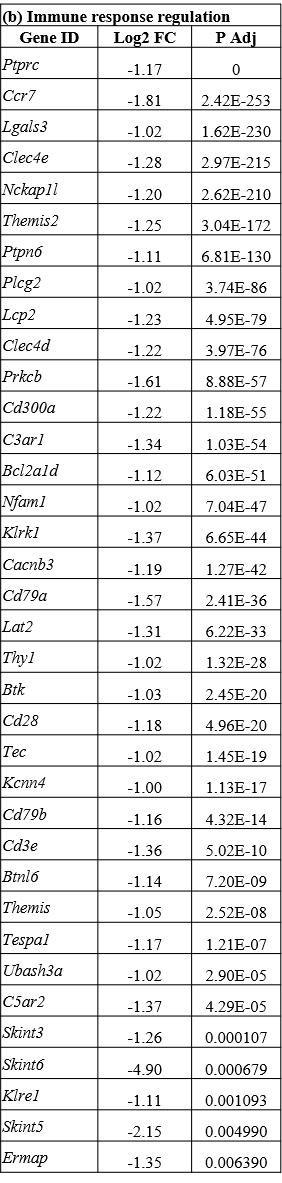


**Supplemental Table 6.** RNA sequencing analysis showed significant upregulation of (a) cytokine receptor activity, and (b-c) chemokine mediated signaling pathway gene expression in male macrophages at 30 DPI.


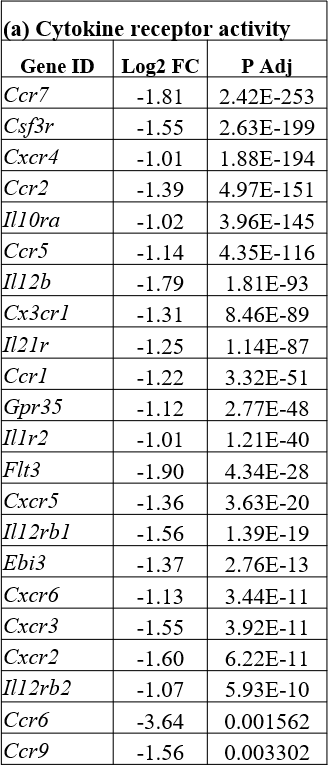

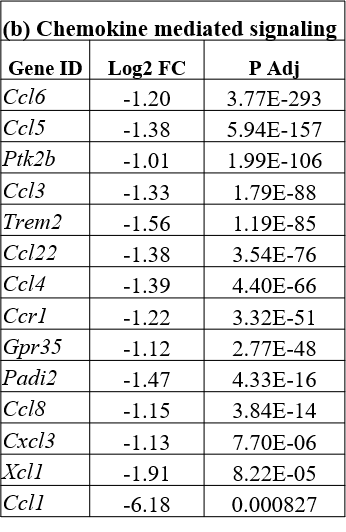

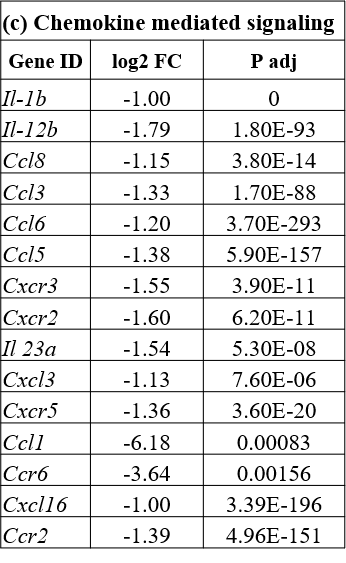

Supplement: Supplemental figures and tables — Figure S1 and Tables S1 to S6. [file spectrum.01790-25-s0001.docx]
